# Are fast food restaurants an environmental risk factor for obesity?

**DOI:** 10.1186/1479-5868-3-2

**Published:** 2006-01-25

**Authors:** Robert W Jeffery, Judy Baxter, Maureen McGuire, Jennifer Linde

**Affiliations:** 1 Division of Epidemiology & Community Health, University of Minnesota School of Public Health, 1300 South 2^nd ^Street, Suite 300, Minneapolis, MN 55454-1015, USA; 2 Guidant Corporation, Cardiac Rhythm Management Group, 4100 Hamline Ave., St. Paul, MN 55112, USA

## Abstract

**Objective:**

Eating at "fast food" restaurants has increased and is linked to obesity. This study examined whether living or working near "fast food" restaurants is associated with body weight.

**Methods:**

A telephone survey of 1033 Minnesota residents assessed body height and weight, frequency of eating at restaurants, and work and home addresses. Proximity of home and work to restaurants was assessed by Global Index System (GIS) methodology.

**Results:**

Eating at "fast food" restaurants was positively associated with having children, a high fat diet and Body Mass Index (BMI). It was negatively associated with vegetable consumption and physical activity. Proximity of "fast food" restaurants to home or work was not associated with eating at "fast food" restaurants or with BMI. Proximity of "non-fast food" restaurants was not associated with BMI, but was associated with frequency of eating at those restaurants.

**Conclusion:**

Failure to find relationships between proximity to "fast food" restaurants and obesity may be due to methodological weaknesses, e.g. the operational definition of "fast food" or "proximity", or homogeneity of restaurant proximity. Alternatively, the proliferation of "fast food" restaurants may not be a strong unique cause of obesity.

## Introduction

Rapid increases in the prevalence of obesity in the US over the last 20 to 30 years have been well documented and their causes much discussed [1-4]. Because of the speed of the change, it has been argued that the cause is more likely to be environmental change, broadly conceptualized to include physical and social factors, than to biological change. Exploration of data on environmental trends has identified a variety of potential environmental contributors. One trend that has attracted particular attention in both the scientific and lay press is the dramatic increase in eating away from home, and particularly at "fast food" outlets. The scientific case for "fast food" restaurants as a causal factor in obesity is based on several observations. First, time trends in eating away from home roughly parallel the national time trends in obesity prevalence [5-10]. Second, although there is no clearly agreed upon definition of the concept, "fast food" outlets have been by far the most rapidly expanding sector of the U.S. food distribution system [9,11,12]. Third, cross-sectional and longitudinal data on self-reported "fast food" restaurant use per se and consumption of foods frequently sold at "fast food" restaurants (e.g. hamburgers and French fries) have been shown to be positively associated with body weight [13-17]. Finally, nutritional analysis of products sold in "fast food" restaurants indicates that they are typically high in energy density, which provides a plausible mechanism through which they might promote excess energy intake [18-20].

Available data on fast food use and obesity are far from conclusive, however. For example, the direction of causation is unclear, i.e., the menus and prices at "fast food" restaurants may result from the demands of an increasingly obese population rather than being a direct cause of obesity. It is also possible that a third variable, such as demographics and lifestyle characteristics (e.g. an aging population with smaller families and a higher percent of two income families), may cause both phenomena.

To date, ecological studies on relationships between obesity and "fast food" restaurant exposure have largely focused on aggregate rather than individual level analysis. There is some evidence that there are more "fast food" restaurants in geographic areas in which obesity prevalence is high (e.g. low income areas) [21-25]. A limited number of studies have also reported positive associations between aggregate measures of food outlet density in defined geographic areas and aggregate measures of obesity in those areas [22-26]. The only published study [27] that we are aware of that assessed relationships between exposure to "fast food" restaurants per se, and ease of access to them at an individual level, however, found no relationship. However, the sample was restricted to low-income children under the age of 5 years. The present investigation attempted to collect data that would bear further on this issue. A sample of individuals from a Midwestern state in the United States was identified and surveyed by telephone to assess body weight and frequency of patronage of "fast food" restaurant. The proximity of their home and work addresses to "fast food" restaurants and "non-fast food" restaurants was estimated using Global Index Systems (GIS) methodology. The association between these variables was examined. The overall hypotheses guiding the investigation was that the proximity of "fast food" restaurants to individuals' homes and/or work settings would be predictive of how often they ate at those restaurants, and also would be predictive of body mass index (BMI) as a measure of obesity. We also examined demographic and behavioral correlates of "fast food" eating.

## Methods

This research was approved by the Institutional Review board of the University of Minnesota. Participants in this investigation were 1,033 residents of the state of Minnesota identified in a random digit-dial telephone survey. Individuals were told the survey was being conducted by the University of Minnesota to identify patterns of eating away from home and attitudes towards it. The survey took approximately 10 minutes to complete. All adults over the age of 18 were eligible to complete the survey. Survey respondents were asked questions about their demographic characteristics, their height and weight, and their eating habits with particular emphasis on frequency of eating away from home. They were also asked for their home and work addresses. The variables used in the current analysis are reported height and weight from which BMI was calculated (kg/m^2^), gender, education, marital status, employment status, household size, number of children, hours of TV watched per week, frequency of reported dieting for weight control, days per week in which individuals reported being physically active for 30 minutes or more, frequency of eating at "fast food" restaurants, and frequency of eating at other restaurants. All of the above were assessed (see Table 2 for sample sizes available for each item) with single item questions. Quality of diet was estimated using a series of questions designed for this study asking people how often in the past week they ate the following high-fat foods: tacos or burritos; hamburgers of cheeseburgers; fried chicken or fried fish; hot dogs, franks, or bratwurst; cold cuts or lunch meats; pizza; fries or onion rings; potato chips, corn chips or popcorn; ice cream of milk shakes; and doughnuts, pastries, cake or cookies. A total fat score was calculated by summing frequencies across items.

Ease of access to restaurants was calculated for each individual using Geographical Index Systems (GIS) methodology. A company specializing in GIS analysis performed the analysis under contract [Claritas, Inc.]. The company used a file of home and work addresses provided by the study in conjunction with public domain databases that have comprehensive listings of food outlets categorized by Standard Industrial Code (SIC). Home and work addresses could not be geo-coded 10.5% and 5.4% of the time, respectively. SIC codes are descriptive labels attached to all businesses in the U.S. by the U.S. government for statistical purposes. The SIC code for eating places is (5812). The study contractor further subdivided eating places into 19 sub-categories using "proprietary" data available to the contractor. Three of these were considered "fast food" restaurants, quick service burger, quick service roast beef, and quick service pizza parlor. Example restaurants meeting these criteria include, McDonald's, Long John Silver's, and Taco Bell. At present, there is no agreed upon definition of "fast food" for research purposes, and other investigators interested in this question have often used all restaurants in analyses rather than "fast food" due to this uncertainty [26]. In the present case we conducted analyses using both the total number of restaurants and the number of "fast food" restaurants as defined by our contractor within circles with radii of 0.5 miles, 1.0 miles and 2.0 miles with home and work addresses as the center of the circles. These distances were based on the expectation that they could be reached by foot or motor vehicle (more likely in the U.S.) in a short period of time.

Food outlet density was defined by the GIS, which calculated number of food establishments. Analyses are conducted using all available data. When data were missing, cases were dropped if all variables in the specific analysis were not present. Two kinds of analyses were performed on the data. Logistic regression analyses were first used to identify variables associated with eating out at "fast food" restaurants one or more times per week compared to fewer times. The second set of analyses examined relationships among body mass index, the number of times per week people reported eating at "fast food" restaurants and other restaurants, and the number of "fast food" or other restaurants within different distances from their home and work addresses. These analyses used linear regression models controlling for age and education, both of which are related to body weight and to place of residence. Initial analyses were stratified by gender. If there were no differences in the relationship by gender, men and women were included in the same analysis with gender as a covariate. The numbers of restaurants at different distances were highly correlated, and the analyses results were very similar for each distance. Analyses results reported here are for the 2-mile radius.

## Results

Table 1 presents descriptive statistics on the study participants. Approximately two-thirds were women. The distribution of reported educational attainment was broad, with approximately one quarter reporting high school education or less and 40% reporting college education or more. The majority of the respondents were married, and most worked outside the home. The average age of respondents was 46 years, average BMI was approximately 26 kg/m^2^, average family size was 2.6 individuals (one of whom was a child), average reported TV hours watched per week was 11.2, physical activity for 30 minutes or more was reported on 3.6 days per week on average, and participants mean reported frequency of dieting to control weight was about midway on a 5 point scale anchored by Always at one end and Never at the other. Reported frequency of fast food eating was similar to that found in previous surveys in this population [13]. The achieved sample was clearly older than the population average, more female, and of higher education. It is believed, however, that there was a fairly broad representation of individuals in the geographic area.

Results of the GIS analysis indicated that on average, there were 39 restaurants of all types within 2 miles of a home address and 94 within 2 miles of a work address. Comparable numbers for "fast food" restaurants were 15 and 32 for home and work site addresses, respectively. The seeming high density of food establishments in this sample may in part be to the fact that about 3/4 of the population of Minnesota is urban. We do not know how these densities compare with those found elsewhere.

Table 2 shows the relationship between demographic and behavioral characteristics and reported eating at "fast food" restaurants at least once per week. Having children in the home was associated with significantly higher rates of reported eating at "fast food" restaurants, as was working outside the home and reporting a higher fat intake. Vegetable intake and frequency of participating in physical activity were inversely related to frequency of reported "fast food" restaurant use.

Table 3 reports the association between body mass index and reported frequency of eating at restaurants. As has been reported by others, we found a significant positive association between BMI and frequency of reported eating at "fast food" restaurants. We found no association between BMI and frequency of reported eating at "non-fast food" restaurants.

Table 4 shows the association between the number of restaurants within 2 miles of individuals' homes and the frequency with which they reported eating at those restaurants. Proximity of "fast food" restaurants to people's home was not significantly related to the frequency with which they reported eating at these restaurants. However, proximity of "non-fast food" restaurants was positively associated with frequency with which people reported eating at those restaurants. Analyses examining similar relationships for work rather than home addresses, we found no relationship for either men or women between reported restaurant eating frequency and proximity of restaurants to work addresses.

The final analyses reported here examined the relationships between body mass index and proximity of restaurants to home and work addresses. We found no relationship between BMI and restaurant proximity to home addresses for either women or men. For men only, however, we found a significant inverse relationship between BMI and restaurant proximity (both "fast" and "non-fast" food). Men with more restaurants close to their places of work were leaner. The relationships for men are shown in Tables 5 and 6.

## Discussion

The results of this investigation provide mixed evidence in regard to the potential effects of a proliferation of convenience food outlets on obesity in the US population. The study showed that how often people report eating at "fast food" restaurants is associated with higher weight and less healthy eating habits. It also showed that working outside the home and having children were associated with higher frequencies of "fast food" restaurant patronage. We also found that the accessibility of restaurants, defined as the number of restaurants within a 2-mile radius of home addresses, was predictive of frequency of reported overall restaurant usage, although not frequency of reported "fast food" restaurant usage. Number of restaurants near people's homes was not associated with BMI. For men only, the number of restaurants near to work addresses was inversely associated with BMI. Overall, therefore, these data seem consistent with the idea that geographic density of restaurants may increase the likelihood that people will eat away from home. We did not, however, find a strong link between obesity itself and restaurant exposure variables.

Given the amount of attention that has been focused on the "fast food" restaurant as a toxic element in our environment that promotes obesity, possible reasons for not finding any relationship between ease of access to such establishments and obesity in our population sample merits additional comment. There are several possible reasons why a real effect might not be detected in this data set. These include the following. The availability of "fast food" restaurant outlets may be relatively homogenous across the U.S. environment or, alternatively, even though different areas have different densities of restaurants, all have enough access that physical access is not a limiting factor governing restaurant patronage. If this is true, addressing the question of whether the number of "fast food" restaurants in an environment are associated with obesity might require a wider range of exposure levels than are possible in a limited geographic environment, e.g. international comparisons.

A second possible reason for failing to find the expected "fast food"/obesity relationship is that the ability to do precise analysis of relationships between "fast food" exposure and obesity may be limited by imprecision in the definition of "fast food" and the definition of "exposure." The prototypical "fast food" outlet is characterized by a relatively limited menu and food preparation options, quick service, paying for meals before they are received, no wait staff, and the option to consume the meal at the restaurant or take it out. However, there are wide variations in these elements in real world settings and which of these, if any, are key is unknown. Some fast food restaurants are also more heavily advertised than others, a factor which also was not taken into account in our definitions. Similarly, defining proximity as a linear distance from a place of residence or place of work may be too simplistic a definition of exposure. Much more germane might be access at particular points in time and space when a person is in a particular need for something to eat (e.g. in route to a child's sporting event). Thus, location of primary domicile or work site may be only one of many variables related to "fast food" restaurant usage. Third, the database available for GIS mapping of food outlets may be sufficiently error prone to make identification of characteristics of particular food outlets from SIC codes problematic. Are the codes up to date, do they actually capture current conditions in every food outlet, who provides the information for the categorization to begin with and how accurate is it? Examination of these questions would require significant additional effort and resources.

Lastly, it needs to be recognized that "fast food" restaurants may not in themselves make a major independent contribution to obesity. If we live lifestyles which are conducive to positive energy balance due in part to excess energy intake, the existence of convenience food outlets may be at least in part the consequence of the way in which lifestyles affect consumer demands for food convenience, palatability and price (e.g. multiple family breadwinners, long work hours and multiple overlapping schedules of family members made possible by increasing affluence) rather than a reaction to industry marketing activities.

### Conclusions

Reported eating at fast food restaurants was associated with having children, with poorer eating and exercise habits and with higher BMI. This study was unable to find a relationship between any measure of restaurant proximity and BMI.

**Table 1:** Characteristics of Survey Respondents

**Categorical Variables**	**N = 1033**	**%**			
Gender (% female)	1033	68.6			
Education (%):	1030				
≤ High school		24.7			
Vocational/Some college		33.4			
≥ College		41.9			
Marital status (% married)	1026	68.5			
Work outside home (% yes)	1031	63.5			
Eat fast food ≥ 1/wk (% yes)	1031	51.4			
					
**Continuous Variables**	**N**	**Mean**	**SD**	**Minimum**	**Maximum**

Age (y)	958	46.2	14.8	18.0	76.0
BMI	985	25.5	4.6	14.3	55.6
Household size (persons)	1029	2.6	1.4	1.0	10.0
Children	793	0.9	1.3	0.0	8.0
TV (hr/wk)	1021	11.2	9.8	0.5	41.0
Physical activity (30-min times/wk)	713	3.6	2.1	0.0	15.0
Frequency of dieting (1 = always, 5 = never)	1027	2.9	1.5	1.0	5.0

**Table 2:** Factors Associated with Fast Food Eating* (≥ one time per week)

	**N**	**Odds ratio**	**CI of odds ratio**	**P**
Married	949	1.105	0.82–1.49	0.51
Any children	759	1.875	1.36–2.59	0.001
Number of children	759	1.137	1.002–1.29	0.05
Household size	952	1.067	0.97–1.18	0.20
Work outside home	952	1.319	0.97–1.79	0.08
Any physical activity (yes)	953	0.695	0.51–0.95	0.02
Physical activity/week	682	0.916	0.85–0.99	0.03
TV (hr/wk)	942	1.014	0.999–1.03	0.07
Frequency of dieting	951	1.027	0.94–1.13	0.58
Fat summary score	954	1.128	1.09–1.16	0.001
Fruit	947	0.945	0.86–1.05	0.27
Vegetables	947	0.837	0.75–0.93	0.001

* Adjusted for age, education, and gender.

**Table 3:** Associations between BMI and Reported 'Fast' and Other Food Restaurant Use*

**Independent variable **(times/wk ≥ 1 vs. none)	**N**	**P value**	**β coefficient**
'Fast' food restaurant use	911	0.02	0.301
Other restaurant use	913	0.71	-0.034
Total restaurant use	911	0.25	0.084

*Adjusted for age, education, and gender.

**Table 4:** Associations of Reported Fast Food and Other Restaurant Use (per week) with Numbers of Restaurants Within 2 Miles of Home*

**Independent variable**	**Dependent variable**	**N**	**β coefficient**	**P value**
Number of fast food restaurants	Frequency of fast food restaurant use/wk	911	0.301	0.01
Number of other restaurants	Frequency of other restaurant use/wk	911	-0.034	0.71
Total number of restaurants	Frequency of any restaurant use/wk	913	0.083	0.24

*Adjusted for age, education, and gender.

Address failure

10.5% of home addresses failed to match (of 1030)

5.4% of work addresses failed to match (of 278)

**Table 5:** Associations Between BMI and Numbers of Restaurants Within 2 Miles of Home in Men*

**Independent variable**	**Dependent variable**	**N**	**β coefficient**	**P value**
Number of fast food restaurants	BMI	305	-0.011	0.20
Number of other restaurants	BMI	305	-0.008	0.27
Total number of restaurants	BMI	305	0.002	0.26

*Adjusted for age and education (*results for Females are NS*)

**Table 6:** Associations Between BMI and Numbers of Restaurants Within 2 Miles of Work in Men*

**Independent variable**	**Dependent variable**	**N**	**β coefficient**	**P value**
Number of fast food restaurants	BMI	95	-0.029	0.008
Number of other restaurants	BMI	95	-0.022	0.01
Total number of restaurants	BMI	95	-0.005	0.01

*Adjusted for age and education (*results for Females are NS*)
