# Correction: Are fast food restaurants an environmental risk factor for obesity?

**DOI:** 10.1186/1479-5868-3-35

**Published:** 2006-10-12

**Authors:** Robert W Jeffery, Judy Baxter, Maureen McGuire, Jennifer Linde

**Affiliations:** 1 Division of Epidemiology & Community Health, University of Minnesota School of Public Health, 1300 South 2^*nd *^Street, Suite 300, Minneapolis, MN 55454-1015, USA; 2 Guidant Corporation, Cardiac Rhythm Management Group, 4100 Hamline Ave., St. Paul, MN 55112, USA

## Correction

Following the publication of this article [1], it was recognized that two sections had been omitted, and that the tables displayed incorrect statistical data, which did not correspond with the data described within the text.

The corrections are as follows,

Following the discussion section of the article, the following conclusions should have been included, "Reported eating at fast food restaurants was associated with having children, with poorer eating and exercise habits and with higher BMI. This study was unable to find a relationship between any measure of restaurant proximity and BMI."

Following the conclusion section, we would like to make the following Acknowledgements, "This research was supported by Grant DK50456 from the National Institute of Diabetes and Digestive and Kidney Diseases. We wish to acknowledge Claritas, Inc. for their assistance with GIS coding."

Tables 3 and 4 contained data that did not represent the results, or the conclusions that were drawn, from this study.

The corrections to table 3 are as follows,

Row "'Fast' food restaurant use," 911 within the column "N" should be changed to 913.

Row "Total restaurant use," 911 within the column "N" should be changed to 913

The corrected table 3 appears as (see table 1).

**Table 1:** Correction:Table 3: Associations between BMI and Reported 'Fast' and Other Food Restaurant Use*

**Independent variable ** (times/wk ≥ 1 vs. none)	**N**	**P value**	**β coefficient**
'Fast' food restaurant use	913	0.02	0.301
Other restaurant use	913	0.71	-0.034
Total restaurant use	913	0.25	0.084

*Adjusted for age, education, and gender.

The corrections to table 4 are as follows,

Within the row "Frequency of fast food restaurant use/wk," 911 within column "N" should be 954. Also, within this row, 0.301 within column "β coefficient" should be 0.001, and 0.01 within column "P value" should be 0.42

Within the row "Frequency of other restaurant use/wk," 911 within "N" column should be 955. Also within this row, -0.034 within the column "β coefficient" should be 0.001, and 0.71 within the column "P value" should be 0.02

Within the row "Frequency of any restaurant use/wk," 913 in "N" column should be 953. Also within this row, 0.083 within "β coefficient" column should be 0.001, and 0.24 within column "P value" should be 0.08

The corrected table 4 appears as (see table 2).

**Table 2:** Correction:Table 4: Associations of Reported Fast Food and Other Restaurant Use (per week) with Numbers of Restaurants Within 2 Miles of Home*

**Independent variable value**	**Dependent variable**	**N**	**β coefficient**	**P**
Number of fast food restaurants	Frequency of fast food restaurant use/wk	954	0.001	0.42
Number of other restaurants	Frequency of other restaurant use/wk	955	0.001	0.02
Total number of restaurants	Frequency of any restaurant use/wk	953	0.001	0.08

*Adjusted for age, education, and gender.

Address failure

10.5% of home addresses failed to match (of 1030)

5.4% of work addresses failed to match (of 278)

## References

[B1] Jeffery RW, Baxter J, McGuire M, Linde J (2006). Are fast food restaurants an environmental risk factor for obesity?. Int J Behav Nutr Phys Act.

